# Methylation and polygenic risk scores capture different features of Systemic Lupus Erythematosus

**DOI:** 10.1093/rheumatology/keag359

**Published:** 2026-07-10

**Authors:** Holme Vestin, Nina Oparina, Maija-Leena Eloranta, Elisabeth Skoglund, Ioanna Giannakou, Martina Frodlund, Iva Gunnarsson, Christopher Sjöwall, Elisabet Svenungsson, Lars Rönnblom, Juliana Imgenberg-Kreuz, Dag Leonard

**Affiliations:** Department of Medical Sciences, Rheumatology, Uppsala University, Uppsala, Sweden; Department of Medical Sciences, Rheumatology, Uppsala University, Uppsala, Sweden; Department of Medical Sciences, Rheumatology, Uppsala University, Uppsala, Sweden; Department of Medical Sciences, Rheumatology, Uppsala University, Uppsala, Sweden; Department of Medical Sciences, Rheumatology, Uppsala University, Uppsala, Sweden; Department of Biomedical and Clinical Sciences, Division of Inflammation and Infection/Rheumatology, Linköping University, Linköping, Sweden; Division of Rheumatology, Department of Medicine, Karolinska Institutet, Karolinska University Hospital, Stockholm, Sweden; Department of Biomedical and Clinical Sciences, Division of Inflammation and Infection/Rheumatology, Linköping University, Linköping, Sweden; Division of Rheumatology, Department of Medicine, Karolinska Institutet, Karolinska University Hospital, Stockholm, Sweden; Department of Medical Sciences, Rheumatology, Uppsala University, Uppsala, Sweden; Department of Medical Sciences, Rheumatology, Uppsala University, Uppsala, Sweden; Department of Medical Sciences, Rheumatology, Uppsala University, Uppsala, Sweden

**Keywords:** systemic lupus erythematosus, epigenome, DNA methylation, genetics, interferon

## Abstract

**Objectives:**

The aetiopathogenesis of SLE encompasses genetic, environmental and epigenetic factors. We investigated associations between an SLE methylation risk score (MRS), HLA-DRB1*03:01, a non-HLA polygenic risk score (PRS), and clinical and immunological phenotypes.

**Methods:**

DNA methylation in whole blood from patients fulfilling ≥4 ACR-82 criteria and from controls was investigated using the Illumina HM450K array. The discovery cohort included 311 patients and 400 controls, and the replication cohort comprised 175 patients and 187 controls. Seventeen independent, top differentially methylated CpG sites (Δβ of ≥0.1) from case–control comparisons were used to calculate the MRS. Genotyping was performed using the Immunochip, and the PRS included 57 non-HLA SLE single-nucleotide variants. Clinical data were collected from patient charts, and serum IFN-α2 was measured using Simoa.

**Results:**

Higher MRS was strongly associated with serum IFN-α2 levels (*P* = 1.04 × 10^−14^). In both cohorts, higher MRS was associated with DLE, immunologic involvement, and anti-SSA/SSB/RNP/Sm autoantibodies (all *P* < 0.05), and with higher disease activity in the discovery cohort (*P* = 1.50 × 10^−4^). MRS was also elevated in patients with multiple autoantibodies (*P* < 1.0 × 10^−15^) and in HLA-DRB1*03:01 carriers (*P* < 1.0 × 10^−3^). In contrast, higher PRS was associated with nephritis, anti-dsDNA positivity, and lower prevalence of anti-SSB antibodies (all *P* < 0.05). No correlation was observed between the MRS and the PRS (*P* = 0.35).

**Conclusion:**

The MRS defines an IFN-high, HLA-DRB1*03:01-linked SLE subset with multiple autoantibodies, partly distinct from PRS-associated nephritis risk, highlighting potentially divergent pathogenic pathways. These findings underscore the value of integrating genetic and epigenetic data to better understand underlying disease mechanisms in SLE.

## Graphical abstract

**Graphical Abstract:**
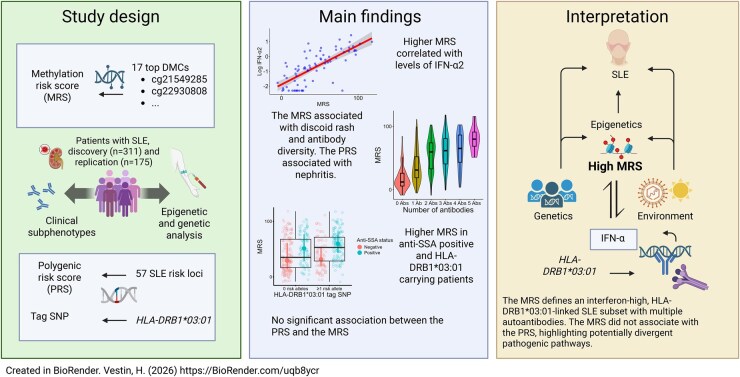

Rheumatology key messages

- Higher MRS, but not PRS, was correlated with increased levels of serum IFN-α.
- The MRS was associated with discoid rash, haematologic disorder, hypocomplementemia, and antibodies including anti-SSA and HLA-DRB1*03:01.
- Higher PRS was linked to nephritis and anti-dsDNA positivity, but was not associated with the MRS.

## Introduction

SLE is an autoimmune, heterogeneously presenting disease characterized by dysregulated innate and adaptive immune responses [1]. The disease is polygenic, although there are monogenic variants that contribute to disease in rare cases [2]. So far, 330 genomic susceptibility loci associated with SLE have been identified [3, 4], and a high polygenic risk score (PRS) has been demonstrated to predict more severe disease phenotypes [5]. The concordance rate between monozygotic twins has been reported to be 24% to 69% [6], and the heritability has been estimated at 44% [7].

In addition to genetic status, epigenetic modifications together with environmental exposures (including hormonal factors) act in a complex interplay ultimately precipitating the immunological aberrations observed in SLE [8]. Epigenetic changes principally include DNA methylation, histone modifications and non-coding RNAs [9]. DNA methylation occurs when a methyl group is added to the 5ˈ carbon on the cytosine residue in CpG sites, which are dinucleotides composed of cytosine, phosphate and guanine in a 5ˈ to 3ˈ direction. This covalent modification typically causes transcriptional silencing through inhibition of transcription factor binding [10].

DNA methylation is subject to genetic control. In the same cohort as the present study, Imgenberg-Kreuz *et al.* reported genetic regulation in the form of cis-methylation quantitative trait loci (meQTL) at 466 (16%) differentially methylated CpG sites (DMCs) in patients with SLE [11]. Further, it was found that 7254 out of 385 851 CpG sites included in the Illumina HM450K array were differentially methylated in patients with SLE, with 75% being hypomethylated and the largest effects being observed in IFN-regulated genes.

Interestingly, IFN-α, a type I IFN, has been shown to induce expression of HLA-DRB1 in lupus T cells [12]. The HLA-DRB1*03:01 risk allele codes for an epitope that, in the presence of IFN-γ, has been reported to elicit transcriptomic programs that are characteristic of SLE and to alter cellular responses in an SLE-like manner [13]. The HLA-DRB1*03:01 allele is part of an extended risk haplotype that is in strong linkage disequilibrium with *C4A* copy number, and we have shown that lower *C4* copy number is associated with SLE and anti-SSA/SSB antibodies [14].

We hypothesized that a standardized procedure of quantifying the DNA methylation changes at top DMCs in patients with SLE can be used to predict clinical presentation. The aim of the study was to investigate associations between a SLE methylation risk score (MRS) and clinical variables, and to delineate whether the phenotypic panorama defined by an elevated MRS can be distinguished from the clinical presentation linked to a high PRS. Further, we aimed to analyse the interrelations between the PRS and the MRS, across HLA-DRB1*03:01–positive and –negative patients.

## Method

### Subjects and samples

The discovery cohort included 311 patients with SLE from Uppsala and Linköping University hospitals and a control group of 400 healthy blood donors visiting the Department of Transfusion Medicine, Uppsala University Hospital. The replication cohort comprised 175 patients from Karolinska University Hospital and 187 population controls [11]. All patients fulfilled the ACR-1982 classification criteria for SLE [15]. Controls were matched for age and sex. Clinical data were collected from patient charts and included sex, age at sampling, ACR-82 criteria [15], autoantibodies (anti-SSA, anti-SSB, anti-RNP, anti-Sm and anti-dsDNA), disease activity measured as either SLEDAI 2000 (SLEDAI-2K) [16] or SLAM [17], and medications at sampling. Simoa® was used to assess IFN-α2 concentration in serum (Supplementary Data S1) [18].

### DNA methylation analysis

Patient and control whole-blood DNA was subjected to Illumina HumanMethylation 450k BeadChip methylation array analysis, using a microarray measuring DNA methylation levels at individual CpG sites on bisulfite-converted DNA and thereby generating methylation data for various regions across the genome [19]. For detailed methodological information, see Imgenberg-Kreuz *et al.* (2018) [11]. The DNA methylation analysis included analysis of methylation levels of 487 577 CpG sites in whole-blood genomic DNA from patients and controls. Subsequently, signal intensity data were exported to the Minfi R package for quality control (QC) and Subset-quantile Within Array Normalisation (SWAN), generating a post-QC dataset including 385 851 CpG sites.

### Methylation analysis and development of the MRS

The MRS was calculated based on DNA methylation levels at previously published DMCs of patients with SLE compared with controls, identified in an epigenome-wide association study adjusted for estimated relative leucocyte composition (Imgenberg-Kreuz *et al.* [11]). Significant DMCs with an effect size (β value) of ≥0.1, located at independent genetic loci, were selected, rendering a total of 17 CpG sites (Table 1).

**Table 1:** CpG sites included in the methylation risk score (MRS)

				Discovery		Replication	
	Gene annotation	Hypomethylated in SLE	IFN induced	*P* value	Methylation Δβ	*P* value	Methylation Δβ
cg21549285	*MX1* ^a^	Yes	Yes	3.5 × 10^−139^	−0.42	6.4 × 10^−83^	−0.47
cg22930808	*PARP9*	Yes	Yes	1.4 × 10^−^^105^	−0.27	1.2 × 10^−74^	−0.33
cg05696877	*IFI44L* ^a^	Yes	Yes	1.9 × 10^−120^	−0.26	4.1 × 10^−86^	−0.32
cg05552874	*IFIT1* ^a^	Yes	Yes	2.5 × 10^−128^	−0.25	1.8 × 10^−73^	−0.27
cg06981309	*PLSCR1*	Yes	Yes	4.9 × 10^−157^	−0.24	7.6 × 10^−91^	−0.25
cg23570810	*IFITM1* ^a^	Yes	Yes	1.6 × 10^−5^	−0.20	3.4 × 10^−60^	−0.24
cg01028142	*CMPK2*	Yes	Yes	1.2 × 10^−64^	−0.15	4.5 × 10^−47^	−0.19
cg20098015	*ODF3B*	Yes	Yes	7.1 × 10^−96^	−0.15	1.1 × 10^−52^	−0.16
cg14864167	*PDE7A*	Yes	Yes	3.6 × 10^−41^	−0.14	4.0 × 10^−37^	−0.20
cg17052170	*LOC100133669*, *LY6E*^a^	Yes	Yes	1.5 × 1 0 ^−58^	−0.13	2.6 × 10^−35^	−0.15
cg08926253	*IRF7* ^a^	Yes	Yes	6.8 × 10^−81^	−0.13	1.5 × 10^−54^	−0.14
cg01787084	*FBXO31*	Yes	NA	1.4 × 10^−126^	−0.13	8.3 × 10^−61^	−0.10
cg08450017	*CXCR6* ^a^, *FYCO1*	No	Yes	1.4 × 10^−130^	0.13	1.8 × 10^−51^	0.10
cg03546163	*FKBP5*	Yes	Yes	1.5 × 10^−66^	−0.13	1.8 × 10^−24^	−0.11
cg17608381	*HLA − A* ^a^	Yes	Yes	4.8 × 10^−25^	−0.11	4.1 × 10 ^−15^	−0.12
cg10152449	*CHST12* ^a^	Yes	Yes	3.6 × 10^−102^	−0.11	3.5 × 10^−49^	−0.10
cg01190666	*HELZ2* (*PRIC285*)	Yes	Yes	1.2 × 10^−111^	−0.10	5.7 × 10^−61^	−0.11

a Differentially methylated in the same direction in PRECISESADS (https://bioinfo.genyo.es/precisesadsdata/) [22]. IFN regulation determined by interferome (https://interferome.org/interferome/) [26]. NA, information not available.

The score was calculated according to the formula developed by Feng X *et al.* [20] and described for DNA methylation data by Björk A *et al.* [21]. The mean and s.d. of the methylation β value for each CpG site in the cohort-specific control group were used to achieve standardized values (Z-scores) for each individual:


$$Z-{\mathrm{score}}_{\mathrm{individual}}=(\beta \;{\mathrm{value}}_{\mathrm{individual}}-{\mathrm{mean}}_{\mathrm{control}})/S.D._{\mathrm{control}}$$

The final methylation scores were obtained through summation of the Z-scores. Z-scores of CpG sites known to be hypomethylated in SLE were multiplied by −1. In total, 16 of the 17 CpG sites included in the MRS were hypomethylated. Next, genes annotated to the CpG sites included in the MRS were investigated for differential methylation in data from the PRECISESADS methylation browser (https://bioinfo.genyo.es/precisesadsdata/) [22]. Subsequently, a methylation score based on PRECISEADS-filtered DMCs, normalized against cohort-specific controls, was calculated for each individual.

### Genotyping and polygenic risk score development

In addition to the MRS, an SLE-PRS comprising 57 non-HLA single-nucleotide variants (SNVs), as described by Reid *et al.* was analysed for associations with the same clinical subphenotypes in a combined cohort of patients from the discovery and replication datasets (*n* = 486) [5]. Genotyping was performed using the Illumina 200K Immunochip SNP array, and the PRS was calculated by summation of the weighted risk alleles (Supplementary Data S1). Lastly, genotype data for an HLA-DRB1*03:01 tag SNP (rs1269852) was extracted from the Immunochip dataset [23].

### Association with clinical variables

Associations between the MRS and clinical subphenotypes were first analysed separately in the discovery and the replication cohorts. Subsequently, an MRS normalized to all controls in the combined cohort was calculated for each patient, and analysed for associations with the PRS, the HLA-DRB1*03:01 tag SNP, and clinical parameters.

### Statistical analysis

Multivariable logistic regression models, with age and sex included as covariates, were applied to assess associations between the epigenetic and genetic scores and binary dependent variables. Continuous variables were analysed and compared between independent groups by the Kruskal–Wallis rank sum test with post-hoc Dunn’s test, and by the Wilcoxon rank-sum test when applicable. Linear regression models, adjusted for age and sex, were fitted to assess associations between continuous variables. Statistical analyses were conducted and plots were generated in R studio version 2024.09.1 + 394 [24].

## Results

### MRS distribution in patients and controls

**Figure 1:**
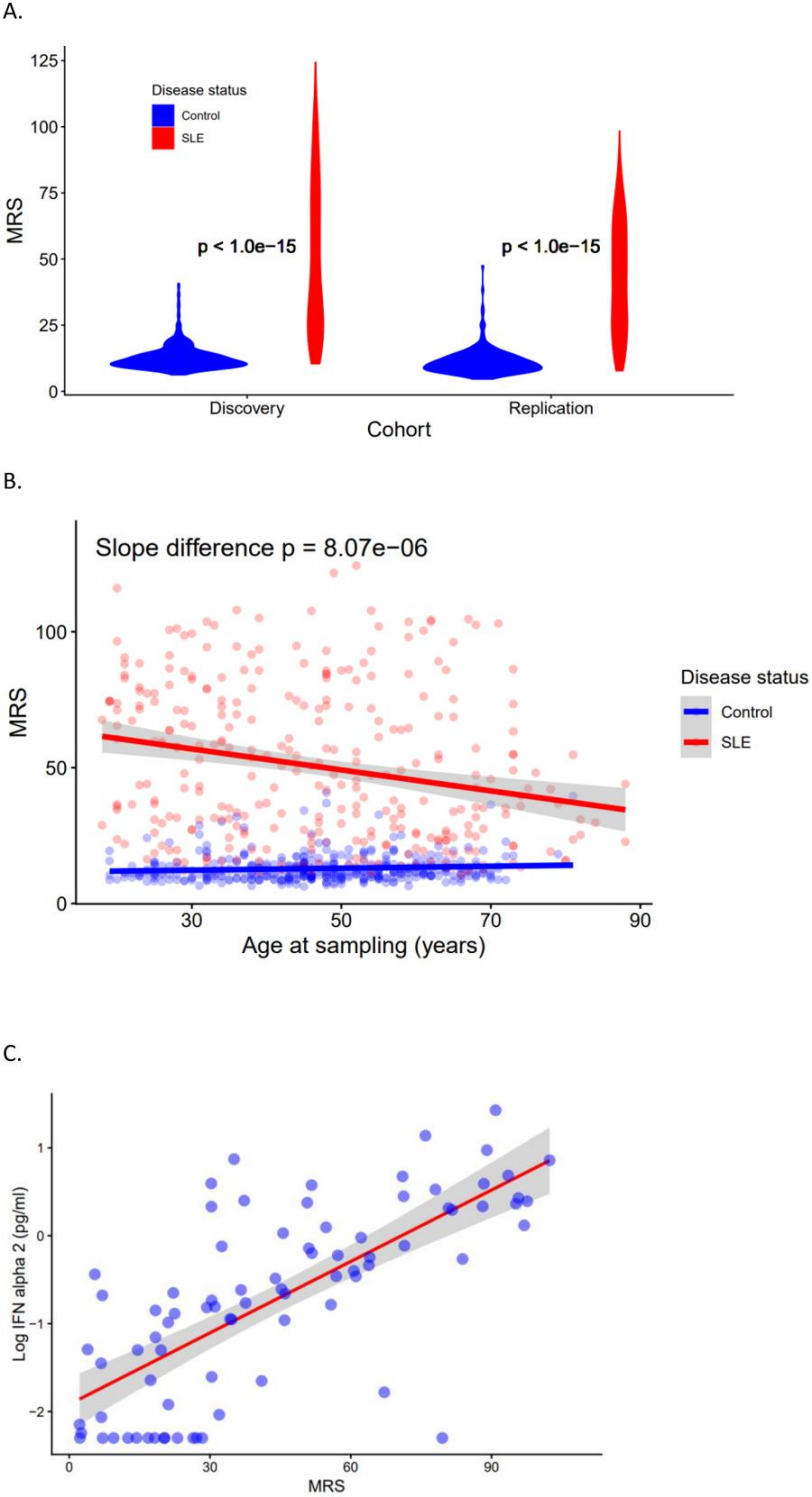
MRS distributions in SLE compared with controls, and across serum levels of IFN-α in a subset of patients. (A) Distribution of the MRS in patients and controls in the discovery and replication cohort. *P*-values, two-sided Mann-Whitney U test. (B) Scatter plot of the relationship between age at sampling and MRS levels across patients and controls in the discovery cohort. *P*-value for difference between the two regression slopes determined by univariate analysis with disease status and age at sampling included with an interaction term. (C) Plot of correlation between the MRS and the common logarithm of IFN alpha 2 (IFN-α2) concentration values (Simoa measurements) [18]. MRS: methylation risk score

The discovery cohort included 311 patients with SLE and a control group of 400 healthy blood donors. The replication cohort comprised 175 and 187 population controls [11]. The methylation score included 17 CpG sites. The largest |Δβ| were observed for the CpG site annotated to *MX1*, followed by *PARP9* and *IFI44L* (Table 1) [11]. Patients with SLE exhibited significantly elevated and more widely distributed MRS values compared with controls in the discovery cohort and also the replication cohort, and an area under the Receiver Operating Characteristic (ROC) curve (AUC) of >0.9 was obtained for the MRS in both the discovery and replication cohorts (Fig. 1A, Fig. S1). In the discovery cohort, male patients with SLE displayed significantly higher MRS scores compared with female patients with SLE (Fig. S1). In the same cohort, older age at the time of sampling was associated with lower MRS in patients with SLE [B = (−0.39), *P* = 3.0 × 10^−5^), whereas a trend towards increased MRS levels was observed in healthy controls with older age at sampling (B = 0.036, *P* = 0.057) (Fig. 1B). The direction of association differed significantly between controls and patients in the discovery cohort as well as in the replication cohort (Fig. 1B and Fig. S2).

### MRS and levels of IFN-α

Since 16 of the 17 annotated genes were found to be IFN induced (Table 1), we examined whether the MRS was associated with serum IFN-α2 levels as measured by Simoa [18]. In a linear regression model adjusted for age at sampling and sex, higher MRS was associated with IFN-α2 levels [Exp(B)  =  1.03, *P* = 1.04 × 10^−14^] (Fig. 1C). Overall, these findings demonstrated that the MRS integrated IFN-induced methylation changes, providing a quantitative measure that could differentiate patients and controls.

### Clinical subphenotypes and MRS

Levels of IFN-α have previously been demonstrated to link with various clinical manifestations of SLE [25]. We therefore examined whether altered MRS predicted specific clinical subphenotypes, employing a discovery and replication cohort design. Higher MRS was associated with an increased risk of DLE, immunologic and haematologic disorder in the discovery cohort, and with discoid rash and immunologic disorder in the replication cohort (Table 2). Higher MRS was also associated with positivity for all the investigated autoantibodies in the discovery cohort, of which anti-SSA/SSB antibodies, anti-RNP antibodies and anti-Sm antibodies were confirmed in the replication data (Table 2). Furthermore, we found an association between increased MRS and higher disease activity and hypocomplementemia in the discovery cohort. However, the association with active disease did not reach statistical significance in the replication cohort, and complement data was not available (Table 2). Among the five treatment categories examined, higher MRS was significantly associated with prednisolone use in both cohorts, whereas lower MRS was linked to ongoing antimalarial treatment, albeit only in the discovery cohort (Table 2).

**Table 2:** Associations between methylation risk score and clinical subphenotypes

	Discovery			Replication		
	*N* (%)	OR (95% CI)	*P*-value	*N* (%)	OR (95% CI)	*P*-value
ACR criteria^a^						
Malar rash	168 (54)	1.00 (1.00–1.01)	3.01 × 10⁻¹	88 (50.3)	1.01 (0.99–1.02)	3.38 × 10⁻¹
Discoid lupus	63 (20.3)	**1.02 (1.01–1.03)**	**2.32 × 10⁻³**	26 (14.9)	**1.02 (1.00–1.05)**	**4.50 × 10⁻²**
Photosensitivity	186 (59.8)	1.01 (1.00–1.02)	1.16 × 10⁻¹	119 (68)	0.99 (0.97–1.01)	1.81 × 10⁻¹
Oral ulcers	61 (19.6)	1.00 (0.99–1.01)	5.65 × 10⁻¹	46 (26.3)	0.99 (0.98–1.01)	5.25 × 10⁻¹
Arthritis	230 (74)	0.99 (0.98–1.00)	5.28 × 10⁻²	146 (83.4)	1.01 (0.99–1.03)	3.44 × 10⁻¹
Serositis	129 (41.5)	1.00 (1.00–1.01)	4.14 × 10⁻¹	76 (43.4)	1.01 (0.99–1.02)	5.16 × 10⁻¹
Nephritis	90 (28.9)	1.01 (1.00–1.01)	2.71 × 10⁻¹	65 (37.1)	1.01 (1.00–1.03)	1.25 × 10⁻¹
Neurologic	18 (5.8)	1.01 (1.00–1.03)	9.00 × 10⁻²	18 (10.3)	1.01 (0.98–1.03)	5.48 × 10⁻¹
Haematologic	186 (59.8)	**1.02 (1.01–1.03)**	**2.02 × 10⁻⁶**	122 (69.7)	1.02 (1.00–1.03)	5.56 × 10⁻²
Immunologic	187 (60.1)	**1.01 (1.00–1.02)**	**1.06 × 10⁻²**	120 (68.6)	**1.02 (1.00–1.04)**	**2.55 × 10⁻²**
Anti-SSA	143 (46.3)	**1.02 (1.01–1.03)**	**1.01 × 10⁻⁵**	79 (45.1)	**1.06 (1.04–1.08)**	**2.49 × 10⁻⁸**
Anti-SSB	85 (27.4)	**1.02 (1.01–1.03)**	**2.67 × 10⁻⁵**	47 (26.9)	**1.04 (1.02–1.06)**	**2.05 × 10⁻⁴**
Anti-RNP	106 (34.2)	**1.03 (1.02–1.04)**	**1.25 × 10⁻⁸**	47 (26.9)	**1.03 (1.01–1.05)**	**5.64 × 10⁻⁴**
Anti-Sm	43 (13.9)	**1.03 (1.01–1.04)**	**2.15 × 10⁻⁵**	24 (13.7)	**1.02 (1.00–1.05)**	**3.62 × 10⁻²**
Anti-dsDNA	180 (58.1)	**1.01 (1.00–1.02)**	**2.32 × 10⁻²**	112 (64)	1.01 (1.00–1.03)	1.85 × 10⁻¹
High disease activity^b^	60 (19.3)	**1.02 (1.01–1.03)**	**1.50 × 10⁻⁴**	78 (44.8)	1.01 (0.99–1.02)	3.52 × 10⁻¹
Low complement^c^	129 (41.5)	**1.01 (1.01–1.02)**	**8.62 × 10⁻⁴**	n.a.	n.a.	n.a.
Treatment						
Prednisolone	192 (61.7)	**1.02 (1.01–1.03)**	**4.21x10^-5^**	98 (56)	**1.02 (1.01–1.04)**	**9.14 × 10^-3^**
Antimalarials	149 (47.9)	**0.99 (0.98–1.00)**	**5.72x10^-3^**	67 (59.8)	1.00 (0.98–1.02)	9.06 × 10⁻¹
AZA	50 (16.1)	1.01 (1.00–1.02)	1.03x10^-1^	34 (25.4)	1.00 (0.98–1.02)	9.42 × 10⁻¹
MMF	34 (10.9)	1.01 (1.00–1.03)	5.39x10^-2^	15 (9.9)	0.99 (0.97–1.02)	7.11 × 10⁻¹
MTX	20 (6.4)	1.02 (1.00–1.03)	5.53x10^-2^	8 (5.4)	0.99 (0.96–1.03)	6.22 × 10⁻¹

Multivariable logistic regression models. *P-*value adjusted for age at sampling and sex are presented with *P* < 0.05 marked in bold.

a Disease manifestations according to the 1982 ACR criteria for SLE (ACR-82).

b High disease activity defined as SLAM > 6 (replication cohort) or SLEDAI > 4 (discovery cohort) [16, 17].

c Hypocomplementemia defined according to SLEDAI [16]. anti-Sm: anti-Smith antibodies.

Given that the epigenetic loci included in the MRS were selected employing a CpG-based procedure, we aimed to investigate whether a score based on CpG sites whose corresponding gene displayed altered methylation in PRECISESADS was associated with similar subphenotypes to those associated with the original MRS. A subset (*n* = 9) of the 17 CpG sites included in the MRS was annotated to a gene that was differentially methylated in the same direction for patients with SLE and for controls, as reported in the PRECISESADS methylation browser (Fig. S3) [22]. The clinical predictability of the score was determined by analysis of the associations with clinical variables in our discovery and replication cohorts. The score was significantly associated with similar clinical subphenotypes to those of the original MRS, including discoid rash, haematologic disorder, anti-SSA/SSB antibodies, anti-RNP antibodies and prednisolone use, and all these associations were significant in both the discovery and replication phases (Table S1).

### MRS and PRS clinical and serological correlates

**Figure 2:**
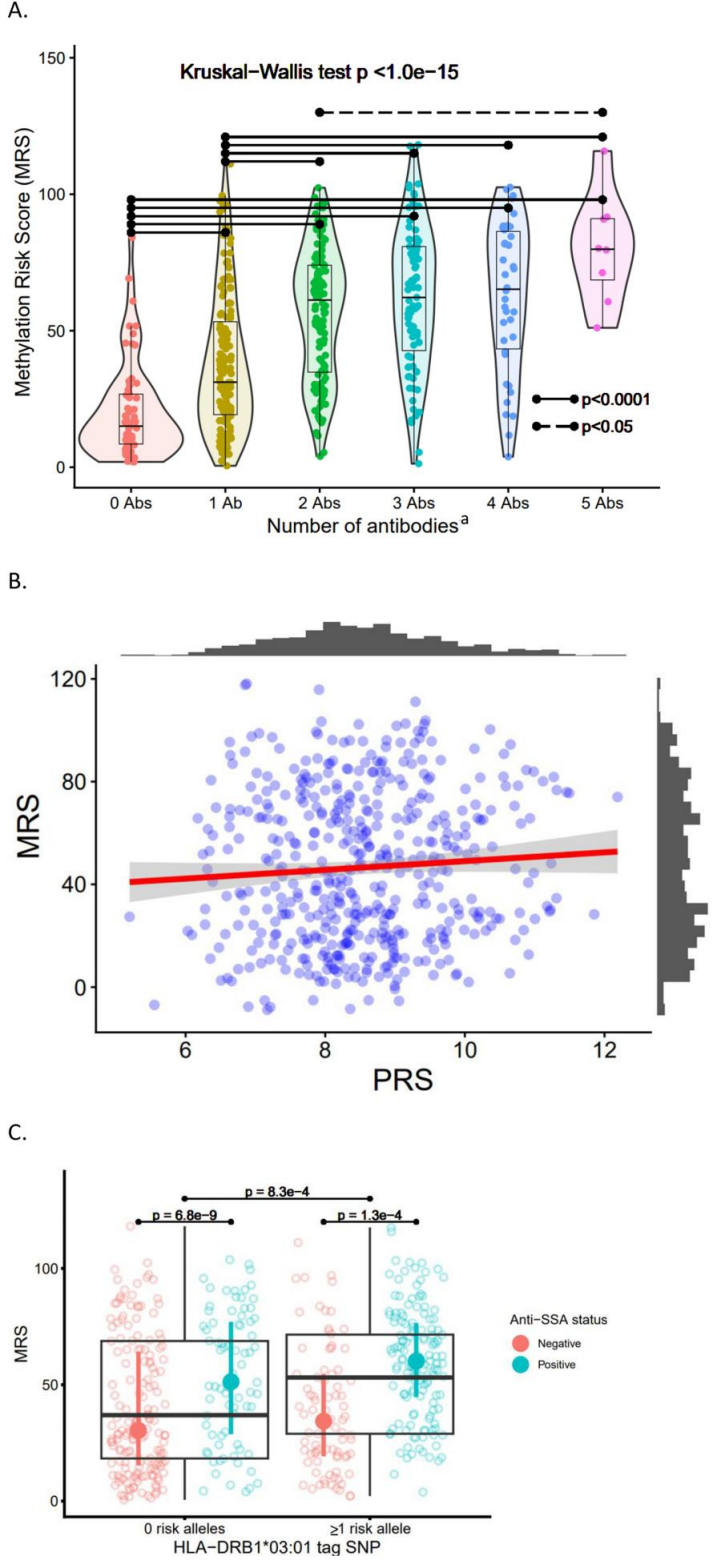
MRS associations with antibodies and the PRS, including stratification by HLA-DRB1*03:01 positivity. (A) Distribution of MRS values in patients with cumulative positivity for different autoantibodies. *P-*values, Kruskal–Wallis rank sum test with post hoc Dunn’s test. *P-*values displayed for all significant differences. (B) Scatter plot with fitted linear regression line of association between the PRS and the MRS. (C) Distribution of the MRS in patients who are non-carriers and carriers of the HLA-DRB1*03:01 risk allele, stratified by anti-SSA antibody status. ^a^Positivity ever for the autoantibodies anti-SSA, anti-SSB, anti-RNP, anti-dsDNA and anti-Sm. MRS, methylation risk score; PRS, polygenic risk score; SNP, single-nucleotide polymorphism

Since Reid *et al.* have previously demonstrated that a higher PRS was associated with a more severe disease, including nephritis and anti-dsDNA antibodies, we proceeded to compare the clinical predictive properties of the MRS and the PRS [5]. To facilitate this comparison, the discovery and replication cohorts were combined, rendering a total of 486 patients with SLE. In this combined cohort, the PRS was associated with nephritis and immunologic disorder, whereas an elevated MRS was linked to a significantly higher risk of DLE, immunologic and haematologic disorder, and high disease activity (Table S2). Among the clinical manifestations, the MRS demonstrated the strongest predictive ability for DLE, with an AUC of 0.67. Further, higher MRS was significantly associated with positivity for all the investigated autoantibodies, with the highest level of significance observed for anti-SSA antibodies. In contrast, higher PRS only conferred increased risk of anti-dsDNA antibody positivity and was associated with lower odds of anti-SSB antibodies (Table S2). Patients with cumulative positivity for multiple autoantibodies exhibited significantly higher MRS levels (Fig. 2A). We further examined whether patients in the top 5% of MRS values exhibited increased risk of any specific clinical subphenotypes. This subgroup demonstrated higher prevalence of malar rash in addition to the previous observed associations (Table S3). Overall, these findings suggest that the MRS captures a broader and to some extent different spectrum of clinical and serological features than the PRS, supporting its potential utility as a complementary biomarker in SLE.

### Minimal overlap between the MRS and PRS

As only two shared associations were found when comparing MRS and PRS associations with clinical findings, we aimed to further explore the relationship between the two scores. In a linear regression model adjusted for age and sex, the scores were not significantly associated (B = 1.04, *P* = 0.35, *R*^2^ = 0.05) (Fig. 2B). Only one gene annotated to a CpG site in the MRS, *IRF7,* was represented in the PRS. The two scores also differed with respect to IFN regulation status. While 16 of the 17 DMCs included in the MRS were annotated to genes reported as IFN-regulated in interferome, only 29 of the 53 genes covered by the PRS were IFN induced (Fig. S4) [26]. Altogether, the methylation pattern and IFN regulation at loci contributing to the PRS differed distinctly from genes captured by the MRS, consistent with the observed lack of association between the two scores.

### MRS and HLA-DRB1*03:01

With reference to the previously known association between the HLA-DRB1*03:01 risk allele and SSA antibodies and the observed link between the MRS and anti-SSA/SSB antibodies in the current study, we compared the MRS and an HLA-DRB1*03:01 tag SNP. Patients carrying at least one HLA-DRB1*03:01 risk allele compared with patients with a non-risk genotype displayed significantly higher MRS scores in the combined cohort (Fig. 2C). Further, patients positive for anti-SSA antibodies exhibited significantly higher MRS levels, which pertained to both risk allele carriers and non-carriers (Fig. 2C). In HLA-DRB1*03:01*–*negative patients, higher MRS conferred a significantly increased risk of nephritis (Table S4). When comparing MRS association with the PRS across HLA-DRB1*03:01 carriers and non-carriers, the slope difference between the two regression lines was significant, with patients without the risk allele exhibiting a tendency towards higher MRS levels with higher polygenic risk (B = 2.9, *P* = 0.064, adjusted for age and sex) (Fig. S5). In summary, we observed a novel association between an HLA-DRB1*03:01 tag SNP and higher MRS, that can be linked to specific SLE features and distinguished from PRS-associated subphenotypes.

## Discussion

In the present study, we investigated associations between MRS and clinical subphenotypes in patients with SLE. Higher MRS was associated with increased levels of IFN-α, discoid rash, multiple autoantibody seropositivity and HLA-DRB1*03:01 carriership. In contrast, a high non-HLA PRS was linked to greater prevalence of renal disease and lower odds of anti-SSB antibodies, suggesting partly different underlying pathogenic mechanisms. We therefore propose that an elevated MRS might confer an epigenetic predisposition for enhanced type I IFN signalling, potentially presenting as distinct clinical subphenotypes linked to HLA-DRB1*03:01.

We report that higher MRS was correlated with higher levels of circulating IFN-α2. Notably, 16 of the 17 CpGs included in the score were hypomethylated, and most annotated genes were IFN-regulated, supporting a close link between the MRS and type I IFN signalling. With regard to methylation stability over time in SLE, Lanata *et al.* reported that a significantly smaller proportion of CpG sites within IFN-responsive genes remained stable longitudinally (53%) compared with CpG sites in non-IFN-related genes (87%)[27]. Among the 20 CpG sites showing the greatest methylation changes over 2 years, four genes (*IFI44L*, *PARP9*, *IFITM1* and *MX1*) overlapped with those included in the MRS. The overall stability of the MRS over time remains to be determined and warrants further longitudinal investigation.

Higher age at sampling was associated with lower MRS in patients, whereas controls displayed the opposite trend. This could be due to medication-associated epigenetic effects, since medications can influence DNA methylation [28, 29]. Alternatively, the epigenetic alterations observed in SLE may attenuate over time. Patients with a late-onset SLE often display a milder disease, which might be related to an age-associated decline in disease expression [30, 31]. In line with this, a recent study reported age-associated epigenetic attenuation of IFN-responsive genes in patients with SLE [32], consistent with the inverse relationship between age and MRS observed of the present study. Taken together, treatment-related epigenetic modulation and age-related hypermethylation represent possible, yet speculative, mechanisms underlying the age-related decrease in MRS levels in patients with SLE. Longitudinal studies investigating the temporal stability of the CpG sites included in the MRS are needed, as the current study only included cross-sectional data.

In the subsequent analyses, higher MRS, but not PRS, was associated with high disease activity and low complement, suggesting that DNA methylation changes may contribute to disease activity. This is consistent with previous observations linking elevated IFN levels to active disease [33], and recent studies reporting DNA hypomethylation of genes, including IFN-regulated genes, in SLE to be associated with disease activity [34, 35]. Higher MRS, but not PRS, was also associated with increased risk of discoid rash, haematologic disorder and multiple autoantibodies, including anti-SSA, anti-RNP and anti-Sm, while patients in the top 5% of MRS values additionally exhibited higher prevalence of malar rash. Indeed, high expression of type I IFN–responsive genes has previously been linked to skin rash, leukocytopenia and autoantibodies [36–38]. Hence, high MRS could reflect an epigenetic susceptibility to type I IFN–driven disease expression, potentially conferring a risk of increased disease expression that may have implications for treatment selection.

Confirming the potential relevance of the genes included in our MRS, the filtered score based on 9 of the 17 CpG sites annotated to genes reported as differentially methylated in SLE showed associations with the same clinical subtypes as the full MRS. Notably, DMCs in three of the genes (*MX1*, *IFI44L* and *IFIT1)* have previously been linked to the same autoantibodies as the MRS [39]. In contrast, a higher PRS conferred a significantly increased risk of renal disease and lower odds of anti-SSB antibody positivity, consistent with our previous findings that a high PRS predicted a more severe disease and greater organ damage accrual [5]. Altogether, the findings suggest that the MRS captures reproducible epigenetic alterations in SLE that define distinct clinical phenotypes from those of the PRS, highlighting its potential as a complementary tool for patient stratification. In a clinical setting, the MRS combined with the PRS could infer prognostic information, with potential implications for the choice of treatment strategies.

A higher MRS was also associated with the HLA-DRB1*03:01 haplotype. This finding, in conjunction with the MRS being associated with discoid rash, SSA/SSB antibodies and haematologic disorder, aligns with the previously described HLA-DRB1*03*–*dominated subgroup of SLE with higher prevalence of anti-SSA/SSB antibodies, skin rash and leukopenia [40]. In addition, Lundtoft *et al.* reported an association between the HLA-DRB*03:01 haplotype, *C4A* copy number variation, and the presence of anti-SSA/SSB antibodies [14]. Collectively, these findings suggest that increased autoantibody production in HLA-DRB1*03:01–positive patients may promote immune complex formation, thereby stimulating type I IFN production. It has been demonstrated that IFN-α, a type I IFN, can induce hypomethylation of genes, including IFN-responsive genes [41, 42]. Hence, sustained IFN exposure could induce DNA methylation changes, further amplifying IFN signalling and contributing to disease expression, including increased disease activity in patients with SLE.

Genetic and epigenetic factors are known to interact in the pathogenesis of SLE. Interestingly, there was no significant correlation between the MRS and the PRS. Only one of the genes connected to the DMCs included in the MRS, *IRF7*, overlapped with the 57 SNPs included in the PRS by Reid *et al.* [5]. However, patients not carrying the HLA-DRB1*03:01 risk allele displayed a tendency towards increased MRS levels, and the regression slope differed significantly from that of HLA-DRB1*03:01–positive individuals. We propose that higher MRS, partly independently of the PRS, associates with a clinical spectrum including HLA-DRB1*03:01 positivity, skin rash, antibody diversity and type I IFN signalling. However, the absence of a correlation between the MRS and the PRS does not necessarily indicate true biological independence, and should be interpreted with caution, as it may reflect limited statistical power or the influence of unmeasured confounding factors. Consistent with this interpretation, in patients lacking the strong pathogenetic contribution of the HLA-DRB1*03:01 haplotype, higher PRS may infer elevated MRS levels. This genetic- and epigenetic-based clinical stratification might be relevant for other HLA-DRB1–associated autoimmune diseases, as patients with SS and patients with SSc display hypomethylation of IFN-induced genes and increased IFN-regulated signalling [38, 43, 44]. Moreover, associations between anti-Ro52 antibodies and the HLA-DRB1*03 allele have been reported in idiopathic inflammatory myopathies, further suggesting that the findings of the current study might be interpretable in a broader autoimmune context [45].

A major strength of the study is the sizes of the cohorts, including a total of 486 patients and 587 controls. Patients were well characterized, and controls were matched for age and sex. Further, a discovery and replication cohort design was employed, increasing the reliability of the findings.

Even though the cohort was comparatively large, the sample size may still be underpowered to identify associations between the risk scores and less common clinical phenotypes. Although adjustment for estimated leucocyte composition was done when identifying DMCs in patients compared with controls, residual confounding by cell type composition cannot be excluded, as bioinformatic correction methods only partially account for this source of variation. Moreover, observed changes in cell type proportions could themselves reflect altered methylation patterns in specific leucocyte subsets. The participants of the present study mainly comprised Caucasians, which limits the generalizability of the findings. Furthermore, the small proportion of male patients included in the study warrants a cautious interpretation of the results in male patients with SLE. Studies including individuals from diverse ancestries, as well as sex-stratified epigenetic analyses, are therefore needed to validate and extend these findings. In addition, similar epigenetic studies in treatment-naïve patients would be valuable, as observed differences in methylation may in part reflect medication-induced epigenetic effects. Finally, the comparison between the methylation and the polygenic risk scores should be interpreted with caution. The PRS applied in this study excluded HLA variants in accordance with established methodology, whereas HLA-DRB1*03:01 was analysed separately. Although this introduces some asymmetry between the genetic and epigenetic measures, it reflects the strong and potentially distinct biological impact of HLA loci, which warrant consideration separately from the non-HLA polygenic burden.

## Conclusion

We report that an SLE MRS, but not a PRS, was associated with discoid rash, haematologic disorder, SSA-antibodies, high disease activity, low complement and elevated serum IFN-α. In contrast, high PRS was linked to nephritis and anti-dsDNA positivity. HLA-DRB1*03:01–positive patients displayed significantly increased MRS levels, and the lack of correlation between the MRS and the PRS suggests that epigenetic and polygenic risk contribute to SLE pathogenesis partly independently. These findings highlight the potential of MRS for identifying an IFN-driven, clinically enriched SLE subset, complementing genetic risk scores in understanding disease heterogeneity.

The study was approved by the Uppsala Regional Ethics Review Board (Dnr: 2009/013 and 2016/155) and was subsequently amended and approved by the Swedish Ethical Review Authority (2020–05065). All participants provided informed consent prior to participation.

## Supplementary Material

keag359_Supplementary_Data

## Data Availability

The data underlying this article will be shared upon reasonable request to the corresponding author.
