# Supplementary material for: Methylation and polygenic risk scores capture different features of Systemic Lupus Erythematosus
Source: Rheumatology (Oxford). 2026 Jul 10;65(8):keag359. doi: 10.1093/rheumatology/keag359 (PMC13452054; doi:10.1093/rheumatology/keag359)
Supplement: Supplementary file 1 [file keag359_supplementary_data.zip › rhe-26-0842-File006.docx]

**Supplementary figures and tables**

A. Discovery cohort

B. B. Replication cohort

**Supplementary Figure S1.** Distribution of the MRS in patients compared to controls across male and female individuals and prediction accuracy of the MRS in the discovery and replication cohort. P-values, two-sided Mann Whitney-U test; AUC, area under the ROC curve.

**Supplementary Figure S2.** Scatter plot of the relationship between age at sampling and MRS levels across patients and controls in the replication cohort. P value for difference between the two regression slopes determined by univariate analysis with disease status and age at sampling included with an interaction term.

**Supplementary Figure S3.** Genes annotated to the CpG sites included in the MRS. Bars indicating level of statistical significance for the CpG sites included in the MRS, and for corresponding genes that were significantly differentially methylated in patients with SLE compared to controls in the PRECISESADS data set (1).

*No available methylation data in PRECISESADS for the specific gene.

| Supplementary Table S1. Associations between the PRECISEADS-based MRS and clinical subphenotypes (1). | | | | | | |
| --- | --- | --- | --- | --- | --- | --- |
|  | **Discovery** | | | **Replication** | | |
| ACR criteria* | **n (%)** | **OR (95% CI)** | **p** | **n (%)** | **OR (95% CI)** | **p** |
| Malar rash | 168 (54) | 1.01 (0.99–1.03) | 2.23×10⁻¹ | 88 (50.3) | 1.02 (0.99–1.06) | 1.55×10⁻¹ |
| Discoid lupus | 63 (20.3) | **1.04 (1.02–1.06)** | **6.49×10⁻⁴** | 26 (14.9) | **1.05 (1.00–1.11)** | **3.91×10⁻²** |
| Photosensitivity | 186 (59.8) | 1.01 (1.00–1.03) | 1.52×10⁻¹ | 119 (68) | 0.98 (0.95–1.02) | 3.78×10⁻¹ |
| Oral ulcers | 61 (19.6) | 1.00 (0.98–1.02) | 7.92×10⁻¹ | 46 (26.3) | 0.98 (0.95–1.02) | 3.50×10⁻¹ |
| Arthritis | 230 (74) | 0.98 (0.96–1.00) | 5.07×10⁻² | 146 (83.4) | 1.03 (0.98–1.07) | 2.57×10⁻¹ |
| Serositis | 129 (41.5) | 1.01 (0.99–1.03) | 3.91×10⁻¹ | 76 (43.4) | 1.02 (0.98–1.05) | 3.20×10⁻¹ |
| Nephritis | 90 (28.9) | 1.01 (0.99–1.03) | 1.72×10⁻¹ | 65 (37.1) | 1.02 (0.99–1.06) | 1.82×10⁻¹ |
| Neurologic | 18 (5.8) | 1.03 (0.99–1.07) | 1.14×10⁻¹ | 18 (10.3) | 1.01 (0.96–1.06) | 7.65×10⁻¹ |
| Hematologic | 186 (59.8) | **1.05 (1.03–1.07)** | **1.09×10⁻⁶** | 122 (69.7) | **1.04 (1.01–1.08)** | **2.22×10⁻²** |
| Immunologic | 187 (60.1) | **1.02 (1.01–1.04)** | **9.20×10⁻³** | 120 (68.6) | 1.03 (1.00–1.07) | 9.58×10⁻² |
| Anti-SSA | 143 (46.3) | **1.05 (1.03–1.07)** | **8.23×10⁻⁷** | 79 (45.1) | **1.13 (1.09–1.18)** | **1.25×10⁻⁸** |
| Anti-SSB | 85 (27.4) | **1.05 (1.03–1.07)** | **3.12×10⁻⁶** | 47 (26.9) | **1.09 (1.05–1.14)** | **7.91×10⁻⁵** |
| Anti-RNP | 106 (34.2) | **1.06 (1.04–1.08)** | **8.05×10⁻⁹** | 47 (26.9) | **1.07 (1.03–1.12)** | **4.53×10⁻⁴** |
| Anti-Sm | 43 (13.9) | **1.06 (1.03–1.09)** | **8.65×10⁻⁵** | 24 (13.7) | 1.04 (0.99–1.10) | 9.06×10⁻² |
| Anti-dsDNA | 180 (58.1) | **1.02 (1.00–1.04)** | **1.92×10⁻²** | 112 (64) | 1.02 (0.98–1.05) | 3.60×10⁻¹ |
| High disease act. ^†^ | 60 (19.3) | **1.04 (1.02–1.07)** | **1.32×10⁻³** | 78 (44.8) | 1.00 (0.97–1.04) | 7.81×10⁻¹ |
| Low compl.^‡^ | 129 (41.5) | **1.03 (1.01-1.05)** | **3.21×10⁻³** | n.a. | n.a. | n.a. |
|  |  |  |  |  |  |  |
| Prednisolone | 192 (61.7) | **1.03 (1.01–1.05)** | **6.64×10⁻⁴** | 98 (56) | **1.04 (1.01–1.07)** | **2.44×10⁻²** |
| Antimalarials | 149 (47.9) | **0.98 (0.96–0.99)** | **9.74×10⁻³** | 67 (59.8) | 1.00 (0.96–1.04) | 9.99×10⁻¹ |
| Azathioprine | 50 (16.1) | 1.02 (1.00–1.04) | 1.06×10⁻¹ | 34 (25.4) | 1.00 (0.96–1.04) | 9.30×10⁻¹ |
| Mycophenolate | 34 (10.9) | 1.02 (1.00–1.05) | 1.01×10⁻¹ | 15 (9.9) | 0.98 (0.92–1.04) | 5.28×10⁻¹ |
| Methotrexate | 20 (6.4) | 1.03 (1.00–1.06) | 8.00×10⁻² | 8 (5.4) | 0.98 (0.91–1.06) | 6.12×10⁻¹ |
|  |  |  |  |  |  |  |

Multivariable logistic regression models. P-values adjusted for age at sampling and sex are presented with p<0.05 marked in bold. Anti-SSA, anti-Sjögren’s-syndrome-related antigen A antibodies; Anti-SSB, anti-Sjögren’s syndrome type B antibodies; Anti-RNP, anti-ribonucleoprotein antibodies; Anti-Sm, anti-Smith antibodies; Anti-dsDNA, anti-double-stranded DNA.

*Disease manifestations according to the American College of Rheumatology 82-criteria for SLE (ACR-82). ^†^High disease activity defined as SLE activity measure (SLAM) >6 (replication cohort) or SLE disease activity index (SLEDAI) >4 (discovery cohort) (2, 3). ^‡^Hypocomplementemia defined according to SLEDAI (2).

| Supplementary Table S2. MRS and PRS associations with clinical subphenotypes. Combined cohort (n = 486). | | | | | | | |
| --- | --- | --- | --- | --- | --- | --- | --- |
|  | **MRS** |  |  |  | **PRS** |  |  |
|  | **n (%)** | **OR (95% CI)** | **AUC** | **p** | **OR (95% CI)** | **AUC** | **p** |
| Malar rash | 256 (52.7) | 1.00 (1.00–1.01) | 0.569 | 2.08×10⁻¹ | 1.02 (0.88–1.19) | 0.548 | 7.56×10⁻¹ |
| Discoid lupus | 89 (18.3) | **1.02 (1.01–1.02)** | **0.666** | **3.51×10⁻⁴** | 0.85 (0.69–1.04) | 0.638 | 1.14×10⁻¹ |
| Photosensitivity | 305 (62.8) | 1.00 (1.00–1.01) | 0.612 | 3.91×10⁻¹ | 0.91 (0.78–1.07) | 0.622 | 2.52×10⁻¹ |
| Oral ulcers | 107 (22) | 1.00 (0.99–1.01) | 0.594 | 9.64×10⁻¹ | 0.96 (0.80–1.16) | 0.594 | 6.68×10⁻¹ |
| Arthritis | 376 (77.4) | 1.00 (0.99–1.00) | 0.521 | 4.46×10⁻¹ | 0.99 (0.82–1.18) | 0.532 | 8.94×10⁻¹ |
| Serositis | 205 (42.2) | 1.00 (1.00–1.01) | 0.572 | 3.64×10⁻¹ | 0.88 (0.75–1.02) | 0.578 | 9.31×10⁻² |
| Nephritis | 155 (31.9) | 1.01 (1.00–1.01) | 0.654 | 5.71×10⁻² | **1.18 (1.00–1.40)** | **0.654** | **4.79×10⁻²** |
| Neurologic | 36 (7.4) | 1.01 (1.00–1.02) | 0.593 | 7.49×10⁻² | 1.12 (0.84–1.49) | 0.570 | 4.27×10⁻¹ |
| Hematologic | 308 (63.4) | **1.02 (1.01–1.03)** | **0.651** | **1.57×10⁻⁷** | 1.03 (0.88–1.20) | 0.545 | 7.34×10⁻¹ |
| Immunologic | 307 (63.2) | **1.01 (1.01–1.02)** | **0.663** | **1.39×10⁻⁴** | **1.40 (1.18–1.67)** | **0.665** | **1.12×10⁻⁴** |
|  |  |  |  |  |  |  |  |
|  |  |  |  |  |  |  |  |
| Anti-SSA | 222 (45.9) | **1.03 (1.02–1.03)** | **0.704** | **1.08×10⁻¹²** | 0.86 (0.73–1.00) | 0.562 | 5.16×10⁻² |
| Anti-SSB | 132 (27.2) | **1.02 (1.02–1.03)** | **0.706** | **2.77×10⁻⁹** | **0.81 (0.68–0.97)** | **0.607** | **2.21×10⁻²** |
| Anti-RNP | 153 (31.5) | **1.02 (1.02–1.03)** | **0.691** | **8.21×10⁻¹¹** | 1.03 (0.88–1.22) | 0.523 | 6.91×10⁻¹ |
| Anti-Sm | 67 (13.8) | **1.02 (1.01–1.03)** | **0.707** | **4.94×10⁻⁶** | 1.05 (0.84–1.31) | 0.618 | 6.52×10⁻¹ |
| Anti-dsDNA | 292 (60.2) | **1.01 (1.00–1.02)** | **0.634** | **2.59×10⁻³** | **1.36 (1.16–1.61)** | **0.649** | **2.72×10⁻⁴** |
| High disease act. ^†^ | 138 (28.5) | **1.01 (1.01–1.02)** | **0.651** | **1.98×10⁻⁴** | 0.93 (0.78–1.10) | 0.609 | 3.96×10⁻¹ |
| Low compl.^‡^ | 129 (41.5) | **1.01 (1.00–1.02)** | **0.663** | **2.20×10⁻³** | 1.12 (0.92–1.37) | 0.632 | 2.60×10⁻¹ |

Multivariable logistic regression models. P-values adjusted for age at sampling and sex are presented with p<0.05 marked in bold. Analysis of AUC values with age and sex included as covariates for both the MRS and the PRS. Anti-SSA, anti-Sjögren’s-syndrome-related antigen A antibodies; Anti-SSB, anti-Sjögren’s syndrome type B antibodies; Anti-RNP, anti-ribonucleoprotein antibodies; Anti-Sm, anti-Smith antibodies; Anti-dsDNA, anti-double-stranded DNA.

*Disease manifestations according to the American College of Rheumatology 82-criteria for SLE (ACR-82). ^†^High disease activity defined as SLE activity measure (SLAM) >6 (replication cohort) or SLE disease activity index (SLEDAI) >4 (discovery cohort) (2, 3). ^‡^Hypocomplementemia defined according to SLEDAI (2).

| Supplementary Table S3. Associations between exhibiting the  5% highest MRS values and clinical subphenotypes. | | | | | |
| --- | --- | --- | --- | --- | --- |
|  |  |  | | |  |
| ACR criteria* | **OR (95% CI)** | | **p** |  |  |
| Malar rash | **2.86 (1.18–8.01)** | | **2.84×10⁻²** |  |  |
| Discoid lupus | 2.00 (0.74–4.86) | | 1.43×10⁻¹ |  |  |
| Photosensitivity | 1.24 (0.53–3.16) | | 6.28×10⁻¹ |  |  |
| Oral ulcers | 1.61 (0.64–3.76) | | 2.86×10⁻¹ |  |  |
| Arthritis | 0.50 (0.22–1.21) | | 1.08×10⁻¹ |  |  |
| Serositis | 1.62 (0.71–3.69) | | 2.46×10⁻¹ |  |  |
| Nephritis | 1.48 (0.61–3.43) | | 3.65×10⁻¹ |  |  |
| Neurologic | 1.15 (0.18–4.13) | | 8.58×10⁻¹ |  |  |
| Hematologic | **3.20 (1.19–11.11)** | | **3.63×10⁻²** |  |  |
| Immunologic | 1.24 (0.53–3.15) | | 6.31×10⁻¹ |  |  |
|  |  | |  |  |  |
| Anti-SSA | 2.14 (0.94–5.16) | | 7.55×10⁻² |  |  |
| Anti-SSB | 1.75 (0.74–3.99) | | 1.87×10⁻¹ |  |  |
| Anti-RNP | **3.49 (1.54–8.23)** | | **3.02×10⁻³** |  |  |
| Anti-Sm | **3.08 (1.19–7.38)** | | **1.42×10⁻²** |  |  |
| Anti-dsDNA | 1.42 (0.61–3.58) | | 4.33×10⁻¹ |  |  |
| High disease act. ^†^ | **2.32 (1.01–5.31)** | | **4.49×10⁻²** |  |  |
| Low compl.^‡^ | **3.61 (1.35–10.79)** | | **1.37×10⁻²** |  |  |

Multivariable logistic regression models. P-values adjusted for age at sampling and sex are presented with p<0.05 marked in bold. Anti-SSA, anti-Sjögren’s-syndrome-related antigen A antibodies; Anti-SSB, anti-Sjögren’s syndrome type B antibodies; Anti-RNP, anti-ribonucleoprotein antibodies; Anti-Sm, anti-Smith antibodies; Anti-dsDNA, anti-double-stranded DNA.

*Disease manifestations according to the American College of Rheumatology 82-criteria for SLE (ACR-82). ^†^High disease activity defined as SLE activity measure (SLAM) >6 (replication cohort) or SLE disease activity index (SLEDAI) >4 (discovery cohort) (2, 3). ^‡^Hypocomplementemia defined according to SLEDAI (2).

**Supplementary Figure S4.** Interferon regulation status of the genes annotated to the risk loci included in the PRS, as reported in the database Interferome (4).

| Supplementary Table S4. Associations between the methylation risk score (MRS) and clinical variables in HLA-DRB1*03:01 negative and positive patients separately. | | | | | | |  |
| --- | --- | --- | --- | --- | --- | --- | --- |
|  | HLA-DRB1*03 :01 negative | | | HLA-DRB1*03 :01 positive | | |  |
|  | **n** | **OR (95% CI)** | **p** | **n** | **OR (95% CI)** | **p** |  |
| ACR-criteria* |  |  |  |  |  |  |  |
| Malar rash | 124 (51.9) | 1.01 (1.00–1.01) | 2.11×10⁻¹ | 122 (53.5) | 1.00 (0.99–1.01) | 6.52×10⁻¹ |  |
| Discoid lupus | 51 (19.8) | **1.02 (1.00–1.03)** | **6.60×10⁻³** | 38 (16.7) | **1.02 (1.01–1.04)** | **4.58×10⁻³** |  |
| Photosensitivity | 158 (161.2) | 1.00 (0.99–1.01) | 7.72×10⁻¹ | 147 (64.5) | 1.01 (1.00–1.02) | 1.31×10⁻¹ |  |
| Oral ulcers | 60 (23.3) | 1.00 (0.99–1.01) | 8.66×10⁻¹ | 47 (20.6) | 1.00 (0.99–1.02) | 6.21×10⁻¹ |  |
| Arthritis | 202 (78.3) | 1.00 (0.99–1.01) | 3.54×10⁻¹ | 174 (76.3) | 1.00 (0.99–1.01) | 9.64×10⁻¹ |  |
| Serositis | 103 (39.9) | 1.00 (0.99–1.01) | 7.51×10⁻¹ | 102 (44.7) | 1.01 (1.00–1.02) | 1.47×10⁻¹ |  |
| Nephritis | 89 (34.5) | **1.01 (1.00–1.02)** | **7.24×10⁻³** | 66 (28.9) | 1.00 (0.99–1.01) | 8.36×10⁻¹ |  |
| Neurologic | 23 (8.9) | 1.01 (0.99–1.02) | 2.57×10⁻¹ | 13 (5.7) | 1.02 (1.00–1.04) | 7.99×10⁻² |  |
| Hematologic | 160 (62.0) | **1.02 (1.01–1.03)** | **1.20×10⁻⁴** | 148 (64.9) | **1.02 (1.01–1.03)** | **7.51×10⁻⁴** |  |
| Immunologic | 166 (64.3) | **1.02 (1.01–1.03)** | **2.80×10⁻⁵** | 141 (61.8) | 1.01 (1.00–1.02) | 2.77×10⁻¹ |  |
|  |  |  |  |  |  |  |  |
| Anti-SSA | 80 (31.1) | **1.02 (1.01–1.03)** | **2.38×10⁻⁴** | 142 (62.6) | **1.03 (1.02–1.05)** | **4.88×10⁻⁸** |  |
| Anti-SSB | 32 (12.4) | **1.02 (1.01–1.04)** | **5.61×10⁻⁴** | 100 (44.1) | **1.02 (1.01–1.03)** | **8.17×10⁻⁵** |  |
| Anti-RNP | 83 (32.2) | **1.03 (1.02–1.04)** | **1.94×10⁻⁸** | 70 (30.8) | **1.02 (1.01–1.03)** | **6.92×10⁻⁴** |  |
| Anti-Sm | 40 (15.5) | **1.03 (1.02–1.04)** | **1.80×10⁻⁵** | 27 (11.9) | **1.02 (1.00–1.03)** | **4.66×10⁻²** |  |
| Anti-dsDNA | 154 (59.7) | **1.02 (1.01–1.03)** | **1.26×10⁻³** | 138 (60.8) | 1.00 (0.99–1.01) | 4.17×10⁻¹ |  |
| High disease act. ^†^ | 78 (30.4) | **1.02 (1.01–1.03)** | **2.93×10⁻⁴** | 60 (26.3) | 1.01 (1.00–1.02) | 9.34×10⁻² |  |
| Low compl.^‡^ | 77 (45.8) | **1.02 (1.01–1.03)** | **4.85×10⁻³** | 52 (36.4) | 1.01 (1.00–1.02) | 1.13×10⁻¹ |  |

Multivariable logistic regression models. P-values adjusted for age at sampling and sex are presented with p<0.05 marked in bold. HLA-DRB1*03:01 negative, no risk allele at the rs1269852 SNP. HLA-DRB1*03:01 positive, homo- or heterozygosity for the risk allele at the rs1269852 SNP. Anti-SSA, anti-Sjögren’s-syndrome-related antigen A antibodies; Anti-SSB, anti-Sjögren’s syndrome type B antibodies; Anti-RNP, anti-ribonucleoprotein antibodies; Anti-Sm, anti-Smith antibodies; Anti-dsDNA, anti-double-stranded DNA.

*Disease manifestations according to the American College of Rheumatology 82-criteria for SLE (ACR-82). ^†^High disease activity defined as SLE activity measure (SLAM) >6 (replication cohort) or SLE disease activity index (SLEDAI) >4 (discovery cohort) (2, 3). ^‡^Hypocomplementemia defined according to SLEDAI (2)

**Supplementary Figure S5.** Scatter plot with fitted linear regression lines of association between the PRS and the MRS across HLA-DRB1*03:01 risk allele carriers and non-carriers. MRS, Methylation Risk Score; PRS, Polygenic Risk Score.

**References**

1.	Barturen G, Babaei S, Català-Moll F, Martínez-Bueno M, Makowska Z, Martorell-Marugán J, et al. Integrative Analysis Reveals a Molecular Stratification of Systemic Autoimmune Diseases. Arthritis & Rheumatology. 2021;73(6):1073-85.

2.	Gladman DD, Ibañez D, Urowitz MB. Systemic lupus erythematosus disease activity index 2000. J Rheumatol. 2002;29(2):288-91.

3.	Liang MH, Socher SA, Roberts WN, Esdaile JM. Measurement of systemic lupus erythematosus activity in clinical research. Arthritis & Rheumatism. 1988;31(7):817-25.

4.	Rusinova I, Forster S, Yu S, Kannan A, Masse M, Cumming H, et al. Interferome v2.0: an updated database of annotated interferon-regulated genes. Nucleic Acids Res. 2013;41(Database issue):D1040-6.
